# “I Need Someone to Talk to” - Perspectives From Low Income Older Adults in Assisted Living and Feasibility Pilot

**DOI:** 10.1093/geroni/igaf122.1718

**Published:** 2025-12-31

**Authors:** Daniel David

## Abstract

Medicaid-funded assisted living programs (ALPs) provide essential housing and care for low-income older adults in New York City. However, many residents experience significant barriers to serious illness discussions and advance care planning (ACP), including social isolation, mistrust of healthcare providers, and limited access to resources. Findings from 72 qualitative interviews reveal that ALP residents often enter care through medical or social crises, shaping their perspectives on serious illness and end-of-life planning. Many express ambivalence toward ACP due to past healthcare experiences, uncertainty about options, and a lack of trusted discussion partners. However, structured conversations with trusted personnel provide an opportunity to reframe these discussions, empowering residents to make informed decisions aligned with their values. In this symposium we will present qualitative findings from ALP resident perspectives on: 1. Arrival Stories - How did they get to ALP? 2. Addressing Serious Illness Needs 3. Surviving Aging – Finding late-life meaning in ALP 4. Telehealth Ambivalence and Serious Illness Conversations To address these challenges, we developed “Someone to Talk To”, a community-driven pilot initiative integrating trained community health workers (CHWs) under nurse supervision to facilitate structured conversations about serious illness. This program builds on five years of collaboration with ALP residents, incorporating insights from a resident-led community advisory board (CAB), staff and assisted living administration. The CAB emphasized the need for a strengths-based approach that prioritizes autonomy, meaning, and proactive decision-making. We will present preliminary findings of a 3 facility serious illness conversation pilot trial.

